# Effects of polymerized whey protein on survivability of *Lactobacillus acidophilus* LA‐5 during freeze‐drying

**DOI:** 10.1002/fsn3.1130

**Published:** 2019-07-24

**Authors:** Cuina Wang, Mu Wang, Hao Wang, Xiaomeng Sun, Mingruo Guo, Juncai Hou

**Affiliations:** ^1^ Key Laboratory of Dairy Science Northeast Agriculture University Harbin China; ^2^ Department of Food Science, College of Food Science and Engineering Jilin University Changchun China; ^3^ Department of Nutrition and Food Sciences, College of Agriculture and Life Sciences University of Vermont Burlington VT USA

**Keywords:** freeze‐drying, *Lactobacillus acidophilus* LA‐5, maltodextrin, whey protein, yoghurt

## Abstract

Probiotic cultures are commonly freeze‐dried for storage and distribution. However, freeze‐drying and subsequent storage are accompanied by a decline in cell viability. Whey protein (WP) or polymerized whey protein (PWP) was used to protect *Lactobacillus acidophilus* LA‐5 against damage during freeze‐drying process and the subsequent storage. The protection capacity and effects of polymerized whey protein protected freeze‐dried *L. acidophilus* LA‐5 on physiochemical properties of cow and goat milk yoghurts were evaluated in comparison with maltodextrin (MD). The survival rate of *L. acidophilus* LA‐5 after freeze‐drying decreased in the order of MD (80.91%) > PWP (69.86%) > WP (64.89%). The particles of WP‐ and PWP‐based freeze‐dried samples showed an average diameter of about 10 μm, which was significantly higher than that of MD‐based particles (1.5 μm). Both whey protein preparations showed higher protecting effect than MD at high humidity condition during the 180‐day storage. Addition of freeze‐dried *L. acidophilus* LA‐5 with the presence of WP or PWP improved the protein content and decreased spontaneous whey separation and syneresis significantly for both yoghurts. PWP‐protected *L. acidophilus* LA‐5 addition significantly improved the firmness and adhesiveness of the yoghurt. Freeze‐dried *L. acidophilus* LA‐5 mixed with PWP had higher survivability in yoghurts compared with the culture alone at the end of storage. Data indicated that whey protein can be used to protect probiotics during freeze‐drying and may also improve the physiochemical properties of the yoghurt.

## 1 INTRODUCTION

Probiotics are defined as live microorganisms which confer a health benefit on the host when administered in adequate amounts (FAO/WHO, ). They play a major role in maintaining the equilibrium and stability of the enteric microbiota, which aids gastrointestinal functions, including control of nutrient bioavailability and modulation of gastrointestinal immune activity (Savard et al., 2011). The benefits provided by probiotics are increasingly explored in various types of foods (Brinques & Ayub, 2011). Probiotic strains before consumption must survive and retain their functionality during storage. Probiotic microorganisms can survive a long duration in frozen form and thus are commonly freeze‐dried for storage and distribution (Kurtmann, Carlsen, Skibsted, & Risbo, 2009). For freeze‐drying process, the solvent is frozen and removed via sublimation. During this process, both bacterial cell wall and membrane may be damaged due to the osmotic stress and membrane phase transitions (Kurtmann et al., 2009). To ensure the viability of probiotics during freeze‐drying process and subsequent storage, protective agents are often used (Schwab, Vogel, & Gänzle, 2007).

Maltodextrin (MD) is hydrolyzed starch produced by partially hydrolysis of starch with acid or enzymes (Loksuwan, 2007). MD is the most common carbohydrate matrix for encapsulation (Sanchez, Baeza, Galmarini, Zamora, & Chirife, 2013) and has been used to increase viability of lactic acid bacteria during freeze‐drying (Roover, Vandenbranden, Laere, & Ende, 2000). Maltodextrin has the ability to form very viscous glasses (Semyonov et al., 2010). It protects probiotics during freeze‐drying by raising the glass transition temperature and thereby helping the viable cells to reach the glassy phase (Tripathi & Giri, 2014). The bacteria are immobilized in the viscous glass which prevents deteriorative reactions occurring due to low mobility (Semyonov et al., 2010). The subsequent storage stability of probiotic was also improved by entrapment in the amorphous MD microstructure (Galmarini, Baren, Zamora, & Chirife, 2010).

Whey proteins can be heated to form soluble polymerized whey protein (PWP) (Wang, Gao, Zheng, Zhang, & Guo, 2017). Whey protein and polymerized whey protein have been extensively used for protecting probiotic from harsh environment (de Castro‐Cislaghi, Silva, Fritzen‐Freire, Lorenz, & Sant'Anna, 2012). Special emphasis has been given to the spray‐drying and extrusion methods. During spray‐drying, hydrophobic interactions between the cells and exposed hydrophobic portions of whey protein result in cells being embedded within the walls of the capsules (Khem, Small, & May, 2016). Divalent cation‐induced gelation properties of polymerized whey protein make it an ideal wall material for embedding probiotics using extrusion technique (Ainsley et al., 2005). Information about the effectiveness of whey protein in protecting probiotic bacteria during freeze‐drying is very limited. However, whey protein exhibits excellent film‐forming abilities. Film can be formed through electrostatic interactions, hydrogen bonding, and van der Waals forces that occur between the protein chains as the water evaporates (Jooyandeh, 2011).

The objective of this study was to use whey protein (WP) or polymerized whey protein (PWP) to protect *Lactobacillus acidophilus* LA‐5, assessing the protect effect during freeze‐drying and the subsequent storage and the effects on physiochemical properties of yoghurts in comparison with maltodextrin (MD).

## 2 MATERIALS AND METHODS

### 2.1 Materials

*Lactobacillus acidophilus* LA‐5 and starter culture YF‐L811 (a mixture of *Streptococcus thermophiles* and *Lactobacillus delbrueckii* ssp. *bulgaricus*) were purchased from Chr. Hansen. Whey protein isolate (WPI, 92% on dry weight basis) was purchased from Fonterra Co‐Operative Group. Maltodextrin (DE 20) was purchased from Kemai Co., Ltd. de Mann–Rogosa–Sharpe (MRS) and agar were purchased from BD Difco. Anaerogen gas pack and anaerobic indicator were purchased from Oxoid. Inulin was purchased from local Biological Technology Co., Ltd. Cow milk and goat milk were purchased from the local market. Deionized water was obtained using a Milli‐Q deionization reversed osmosis system (Millipore Corp.).

### 2.2 *L. Acidophilus* LA‐5 culture preparation

Fresh cultures were obtained after activation by three successive transfers in MRS broth. Cultures in late‐log phase (10^10^ cfu/ml) were harvested by centrifugation at 3,000 × ***g*** for 10 min (Avanti J‐E, Beckman Coulter), washed with a sterile peptone solution (0.1%, w/v). The final wet cell mass was weighed and dispersed in peptone solution to obtain suspension with 10^10^ cfu/ml cells.

### 2.3 Preparation of freeze‐dried *L. acidophilus* LA‐5

Polymerized whey protein solution was prepared according to the procedure described by Wang et al. (2017). Maltodextrin (MD), whey protein (WP), and polymerized whey protein (PWP) were used as encapsulating materials. Cell suspension was added to each wall material solution (15%, w/v) at the percentage of 5% (w/w) and then mixed aseptically. The mixtures were frozen overnight in an −18°C freezer and then dried with a freeze dryer (ALPHA1‐2, CHRIST Ltd.). The freeze‐dried cakes were milled manually under aseptic conditions and transferred into sterilized dark bottles. Freeze‐dried powders with different protecting materials were abbreviated as MD‐LA, WP‐LA, and PWP‐LA, respectively.

### 2.4 Survival rate of *L. acidophilus* LA‐5 after freeze‐drying

Freeze‐dried bacterial powder (0.1 g) was dissolved in 10 ml sterile saline solution and vortexed for 1 min allowing 20–30 min for dissolution. The cell was counted using plate method with MRS agar medium. The survival rate of bacteria after freeze‐drying was calculated as the following equation:

$$\text{Survival}\;\text{rate}\left(\%\right)=\left(\log\;N/\log\;N_{0}\right)\times 100$$

where *N* is the number of cells released after drying (cfu/ml), and *N*_0_ is the number of free cells (cfu/ml) added to the suspension before freeze‐drying process.

### 2.5 Particle size measurement of freeze‐dried culture

The size distribution of the particles was measured using a Laser Particle Analyzer (Mastersizer 2000, Malvern) with a Hydro 2000SM (A) sampling device. The range of measurement was from 0.02 to 2000 μm.

### 2.6 Storage stability of freeze‐dried *L. acidophilus* LA‐5

Samples (0.1 g) of all freeze‐dried bacteria were placed into a series of Eppendorf tubes with rubber septa and tightly closed. Samples were stored in glass desiccators, and the storage conditions varied in terms of relative humidity (11%/33%/70%), temperature (4°C/25°C), and oxygen or anaerobic. Saturated salt solutions of lithium chloride, magnesium chloride, and potassium iodide were used to provide relative humidity of 11%, 33%, and 70%, respectively. Anaerogen gas pack and anaerobic indicator were used to create anaerobic condition and indicate the absence of oxygen. Each environment was represented as listed in Table 1. Moisture content of the freeze‐dried powder and survivability of *L. acidophilus* LA‐5 were determined at 0, 7, 14, 21, 30, 45, 60, 90, 120, 150, and 180 days. Moisture content was determined by a moisture meter (MJ33, Mettler Toledo). The cell survival was measured using plate method with MRS agar medium.

**Table 1:** Storage conditions for freeze‐dried *Lactobacillus acidophilus* LA‐5 using different protecting material

Group	Relative humidity (%)	Oxygen	Temperature (^o^C)
D/A/Re	0	Anaerobic	4
D/A/Ro	0	Anaerobic	25
D/O/Ro	0	Oxygen	4
L/A/Re	11	Anaerobic	4
M/A/Re	33	Anaerobic	4
H/A/Re	70	Anaerobic	4

Note

D is for dry state where the relative humidity is 0; A is for anaerobic; O is for Oxygen; Re is for refrigerated temperature; and Ro is for room temperature. L, M, and H are for low, medium, and high relative humidity.

### 2.7 Yoghurt samples preparation

Cow or goat milk with added sugar (7%, w/v), inulin (1%, w/v), and pectin (0.3%, w/v) was heated to 80°C and kept for 20 min for the complete dissolution and pasteurization with a magnetic stirrer (IKA Ared, Pedrollo). After heat treatment, the mix was cooled down to 43°C and inoculated with basic starter culture of YF‐L811 (0.01%, w/v%). Freeze‐dried *L. acidophilus* LA‐5 (1%, w/v) with different protecting materials were added to the mix with free *L. acidophilus* LA‐5 (0.03%, w/v) as control. All samples were incubated at 43°C for 4.5 hr. Samples were then stored at 4°C for following analysis and storage studies. Cow/goat milk yoghurt containing free *L. acidophilus* LA‐5 was abbreviated as C‐0 and G‐0. Cow/goat milk yoghurt containing MD‐LA, WP‐LA, and PWP‐LA was abbreviated as C1‐3 and G1‐3, respectively.

### 2.8 Physiochemical properties of cow and goat milk yoghurts

Cow and goat milk yoghurt samples were determined for total solids, protein, fat, ash, and carbohydrate contents according to the methods described by Wang, Gao, Zhang, Wang, and Guo (2015).

All samples were determined for spontaneous whey separation and syneresis. After 12 hr of storage, a cup of set yogurt was taken from the cold room (4°C), weighed, and kept at an angle of approximately 45° to allow whey collection at the side of the cup. A needle was used to siphon the whey from the surface of the sample, and the cup of yogurt was weighed again. The spontaneous whey separation was expressed as the percent weight of the whey over the initial weight of the yogurt sample (Amatayakul, Sherkat, & Shah, 2006). For syneresis determination, yogurt sample was weighed and fermented in a centrifuge tube. After fermentation, sample was centrifuged at 5,000 × ***g*** for 10 min (Avanti J‐E, Beckman Coulter). The liquid supernatant was separated and weighed. The syneresis was calculated as the percentage of the centrifuged whey over the weight of the yoghurt fermented in a centrifuge tube.

Texture profile of all yoghurt samples was measured by a texture analyzer (CT‐3, Brookfield Engineering Laboratories, Inc) using the following parameters: mode: TPA; probe: TA‐11; distance: 30 mm; trigger: 4.5 g; and speed: 1 mm/s.

### 2.9 Changes in pH and probiotic viability of yoghurt during storage

Cow and goat milk yoghurt samples were determined for changes in pH and viability of *L. acidophilus* LA‐5 weekly for a total of 10. The pH values were determined with a pH‐meter (PHS‐3C, Jingke) calibrated at 25°C. *L. acidophilus* LA‐5 was numerated using MRS agar medium with 10% (v/v) maltose (20%, w/v).

### 2.10 Statistical analysis

All treatments were made in triplicates for three trials. All data obtained from analysis were expressed as mean ± standard deviation (S.D.). The significant differences in data between samples and control were calculated using SPSS version 19 (SPSS Inc.). The significance level was set at *p* < 0.01. Data were checked for homogeneity by Leveneǐs test. When the data were homogeneous, analysis of variance (ANOVA) and then a least squared differences (LSD) model were used. All the figures were drawn by Origin 8.0 (Origin Lab Corporation).

## 3 RESULTS AND DISCUSSION

### 3.1 Effects of polymerized whey protein on survival rate of *L. acidophilus* LA‐5 after freeze‐drying

**Figure 1:**
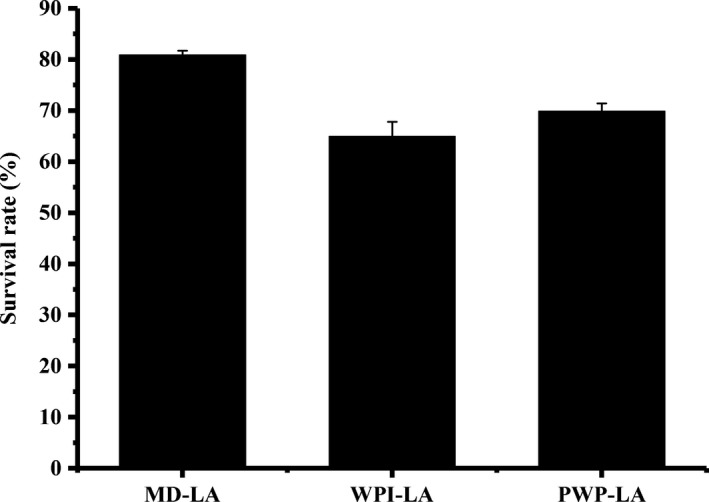
Effects of protecting material on survival rate of *Lactobacillus acidophilus* LA‐5 after freeze‐drying

*Lactobacillus acidophilus* LA‐5, a thermophilic lactic acid bacteria, has shown a balancing activity on the intestinal microecosystem with positive effects on human health (Kim & Gilliland, 1983). It is sensitive to cryogenic treatment which results in structural and physiological injuries that makes its preservation difficult (Murga, Cabrera, Valdez, Disalvo, & Seldes, 2000). Therefore, it is important to protect the probiotic during freeze‐drying. Three protecting materials (MD, WP, and PWP) were examined for their effectiveness in protecting LA‐5 after freeze‐drying, and the results are shown in Figure 1. The protection capacity of the three wall materials expressed by survival rate of LA‐5 decreased in the order of MD (80.91 ± 0.83%) > PWP (69.86 ± 1.54%) > WP (64.98 ± 2.83%). MD‐based freeze‐dried *L. acidophilus* LA‐5 had the highest survival rate (80.91%) among all samples, suggesting that it can protect the probiotic more effectively against low temperature and dehydration. During dehydration conditions, carbohydrates at high concentration will form an amorphous glassy matrix (Kurtmann et al., 2009). *L. acidophilus* LA‐5 can be entrapped into the glassy matrix characterized by high viscosity and low mobility, which is unfavorable for reaction, thus improving the survivability of core material (Ger & Santivarangkna, 2015). PWP protection resulted in higher survival rate (69.86%) than WP (64.98%), indicating that PWP can form a more compact film protecting the LA‐5 from adverse environment. Data indicated that the intrinsic viscosity of heat‐denatured whey protein was much higher than that of native whey protein (Zhang & Vardhanabhuti, 2014) and heat‐denatured whey protein film had higher tensile properties and lower oxygen permeability than native whey protein film (Pérez‐Gago & Krochta, 2001).

### 3.2 Effects of protecting materials on size distribution of *L. acidophilus* LA‐5 powder particles

Results showed that MD‐LA had an average particle size of 1.51 μm (<0.82 μm, 10%; <1.53 μm, 50%; <5.48 μm, 90%), which was significantly lower than those of other two samples (*p* < 0.01). This may be due to the fast dissolution of MD‐LA in water which was used for the measurement medium. WP‐LA and PWP‐LA had average diameters of 8.45 (<1.32 μm, 10%; <8.92 μm, 50%; <10.74 μm, 90%) and 9.07 μm (<1.02 μm, 10%; <10.74 μm, 50%; <14.86 μm, 90%), respectively. PWP‐LA showed the largest particle size may due to the low solubility of polymerized whey protein after heat denaturation (Pelegrine & Gomes, 2012). All the particles had a diameter smaller than 10 μm and can be incorporated in powdered and reconstituted functional foods without affecting the sensory attributes of the food base (Hansen, Allan‐Wojtas, Jin, & Paulson, 2002).

### 3.3 Effects of protecting materials on storage stability of *L. acidophilus* LA‐5

**Figure 2:**
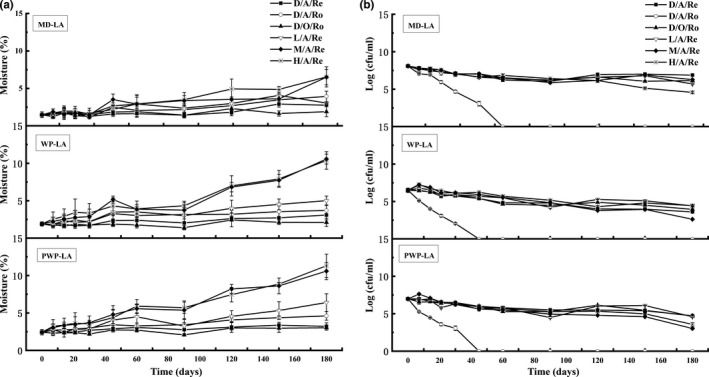
Effects of protecting material on the powder moisture content (a) and survivability of freeze‐dried *Lactobacillus acidophilus* LA‐5 (b) during storage

Freeze‐dried *L. acidophilus* LA‐5 was investigated for stability in terms of moisture content of dried powder and cell survivability during 180‐day storage under various conditions, and the results are shown in Figure 2.

Moisture content of dried power is a critical factor influencing shelf‐life stability of the live bacteria (Meng, Stanton, Fitzgerald, Daly, & Ross, 2008). Figure 2a shows changes in moisture content of all powders stored under various conditions as a function of storage time. MD‐LA showed the lowest initial residual water content (1.49 ± 0.38%) compared with 1.91 ± 0.26% for WP‐LA and 2.44 ± 0.36% for PWP‐LA, respectively. Under conditions of dry state and anaerobic, MD‐LA showed increased moisture contents of 1.28% and 2.45%, which was higher than those of WP‐LA (1.19% and 1.79%) and PWP‐LA (0.77% and 2.18%) at 4°C and 25°C, respectively. The presence of oxygen at dry state and 4°C decreased the water absorption rate for all samples. The moisture increase extent in decreased order is PWP‐LA (0.59%)> MD‐LA (0.41%)> WP (0.2%). Moisture content of all samples was greatly dependent on relative humidity. Whey protein films generally provide poor moisture barriers (Pérez‐Gago, Nadaud, & Krochta, 2010) at high humidity. At lower relative humidity, the amplification for MD‐LA was 1.56%, which was lower than those of WP‐LA (3.1%) and PWP‐LA (3.96%). When the relative humidity was adjusted to 33% and 70%, the water absorption change for MD‐LA increased to about 5% at both conditions. However, it was still lower than those of WP‐LA (8.47% and 8.66%) and PWP‐LA (8.16% and 8.87%) for medium and high humidity, respectively.

Survivability of *L. acidophilus* LA‐5 with different protecting agents during storage is shown in Figure 2b. The viability loss observed for the freeze‐dried probiotic may be attributed to further membrane damage caused by oxidation and lipolysis (Castro, Teixeira, & Kirby, 1996). The stability of probiotic bacteria during storage was greatly affected by storage temperature. Room temperature was one detrimental condition where probiotic decreased sharply and no viable cell can be detected after storage for 60 days (MD‐LA) and 45 days (WP‐LA and PWP‐LA). Even though a longer duration was observed for MA‐LA, it should be noticed that the initial probiotic population for MD‐LA was 8.09 log cfu/ml while those for WP‐LA and PWP‐LA was 6.49 and 6.98 log cfu/ml, respectively. Under refrigerated, dry state, and anaerobic storage condition, MD‐LA had a smaller decline in viability (1.21 logcycle) compared with 2.24 and 2.87 logcycle for WP‐LA and PWP‐LA, respectively. Under condition in the presence of oxygen, PWP‐LA showed a probiotic decrease of 2.28 logcycle which was comparable to that of MD (1.95 log cycle). Humidity is important for dried probiotic, and retention of viability during storage is often enhanced under very low water activity (Meng et al., 2008). MD‐LA had cell declines of 2.4, 1.8, and 3.5 logcycle when stored at conditions of low, medium, and high humidity while those for WP‐LA and PWP‐LA were 2.08, 3.86, and 2.04 and 2.38, 3.95, and 3.41, respectively. Results indicated that whey protein showed advantage over MD in protecting probiotic against loss during storage at conditions of high humidity, which is the common storage condition for freeze‐dried bacterial powder with an opened package.

### 3.4 Effects of freeze‐dried *L. acidophilus* LA‐5 on physiochemical properties of cow and goat milk yoghurts

**Figure 3:**
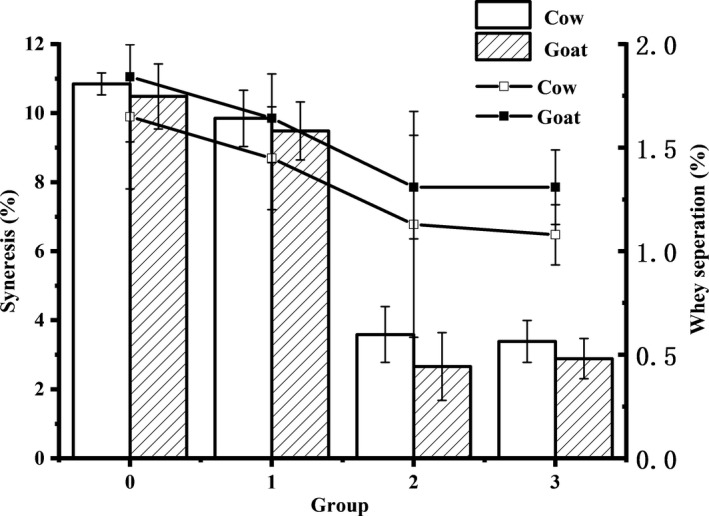
Effects of freeze‐dried *Lactobacillus acidophilus* LA‐5 on whey separation (Line) and syneresis (Bar) of cow and goat milk yoghurts

Fermented dairy products enriched with probiotic bacteria have developed into one of the most successful categories of functional foods. Freeze‐dried *L. acidophilus LA‐5* cultures prepared using different protecting materials were used in symbiotic cow and goat milk yoghurts. Effects of the freeze‐dried *L. acidophilus* LA‐5 on physiochemical properties of yoghurts were investigated, and the results are shown in Table 2 and Figure 3.

**Table 2:** Effects of freeze‐dried *Lactobacillus acidophilus* LA‐5 on chemical compositions (%) and texture profile of cow and goat milk yoghurts

	Total solid	Protein	Fat	Carbohydrate	Ash
C−0	19.31 ± 0.38	2.84 ± 0.04^a^	3.52 ± 0.12	12.25 ± 0.27	0.70 ± 0.00
C−1	19.53 ± 0.61	2.78 ± 0.08^a^	3.30 ± 0.02	12.78 ± 0.14	0.67 ± 0.01
C−2	19.55 ± 0.23	3.68 ± 0.09^b^	3.30 ± 0.09	11.86 ± 0.04	0.71 ± 0.01
C−3	19.58 ± 0.83	3.60 ± 0.16^b^	3.22 ± 0.06	11.99 ± 0.07	0.77 ± 0.00
G−0	19.53 ± 0.35	2.95 ± 0.04^a^	3.32 ± 0.07	12.25 ± 0.08	1.01 ± 0.02
G−1	19.73 ± 0.74	2.89 ± 0.04^a^	3.08 ± 0.03	12.66 ± 0.02	1.10 ± 0.01
G−2	19.83 ± 0.67	3.82 ± 0.11^b^	3.15 ± 0.04	12.10 ± 0.08	1.06 ± 0.02
G−3	19.82 ± 0.22	3.77 ± 0.02^b^	3.13 ± 0.12	11.89 ± 0.09	1.03 ± 0.00

	Firmness (g)	Adhesiveness (mJ)	Cohesiveness	Springiness (mm)
C−0	151.37 ± 22.37^c^	8.18 ± 3.43^b^	0.46 ± 0.08	27.53 ± 1.05
C−1	165.43 ± 28.02^c^	8.42 ± 2.78^b^	0.44 ± 0.04	27.78 ± 0.73
C−2	165.35 ± 33.44^c^	9.05 ± 4.36^b^	0.43 ± 0.03	27.12 ± 0.91
C−3	317.68 ± 7.89^a^	16.51 ± 2.63^a^	0.44 ± 0.01	28.82 ± 1.34
G−0	148.10 ± 5.09^c^	8.50 ± 0.91^b^	0.47 ± 0.04	28.05 ± 0.35
G−1	143.15 ± 10.82^c^	7.44 ± 0.91^b^	0.47 ± 0.01	27.89 ± 0.13
G−2	137.55 ± 9.65^c^	7.41 ± 1.3^b^	0.49 ± 0.02	27.6 ± 0.36
G−3	261.70 ± 15.38^b^	15.75 ± 1.15^a^	0.45 ± 0.02	27.61 ± 1.75

Note

Column with different superscript letters means significant difference at *p* < 0.01.

Table 2 shows the chemical composition (%) of the yoghurt samples. WP‐ and PWP‐based freeze‐dried *L. acidophilus* LA‐5 increased the protein content of cow milk yoghurt to 3.68 ± 0.09% and 3.60 ± 0.16%, which were significantly higher than that of yoghurt containing MD‐LA (2.78 ± 0.08%) and control samples (2.84 ± 0.04%), *p* < 0.01. Goat milk yoghurt samples containing WP‐LA and PWP‐LA showed protein content of 3.82 ± 0.11% and 3.77 ± 0.02%, which was significantly higher than those of goat milk yoghurt containing MD‐LA (2.89 ± 0.04%) and control (2.95 ± 0.04%), *p* < 0.01. Figure 3 shows the spontaneous whey separation percentage and syneresis of cow and goat milk yoghurts. Yoghurts containing WP‐LA or PWP‐LA showed significantly lower whey separation and syneresis values than those containing MD‐LA, *p* < 0.01. PWP‐LA addition significantly improved the firmness and viscosity of yoghurt samples (*p* < 0.01), while WP‐LA and MD‐LA did not affect the texture of yoghurt samples (Table 2).

### 3.5 Effects of freeze‐dried *L. acidophilus* LA‐5 on pH and probiotic population of cow and goat milk yoghurts during storage

**Figure 4:**
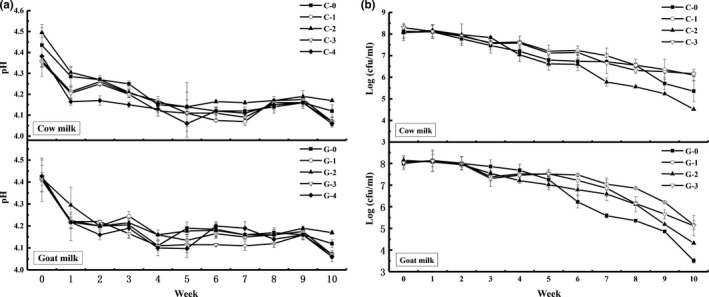
Changes in pH (a) and *Lactobacillus acidophilus* LA‐5 population (b) of cow and goat milk yoghurt samples during a 10‐week storage at 4°C

Dairy products are recognized as the ideal food systems for the delivery of probiotic bacteria to human gut. These products provide probiotic bacteria with a suitable environment in which their growth and viability are promoted (Ross, Fitzerald, Collins, & Stanton, 2002). The dehydrated probiotic products are rehydrated for the revival of cells before consumption. Cow and goat milk yoghurt samples with were monitored for pH and probiotic population changes for a total of 10 weeks, and the results are shown in Figure 4.

Samples with freeze‐dried *L. acidophilus* LA‐5 exhibited a similar pH decreasing trend with control during storage independent of the matrix material used (Figure 4a). Figure 4b shows the changes in *L. acidophilus* LA‐5 population of cow and goat milk yoghurts during a 10‐week storage. An initial high population of about 10^8^ cfu/ml for *L. acidophilus* LA‐5 was observed for both yoghurts regardless of the protecting material type. This indicated that coated *L. acidophilus* LA‐5 can be released into milk matrix and vital metabolic activities of the probiotic were not impaired by freeze‐drying due to the protection effect of materials. The results were consistent with report that cells in powder prepared from water‐soluble materials can be released as soon as free water comes in contact with the microcapsules (Heidebach, Först, & Kulozik, 2010). During storage, viability of *L. acidophilus* LA‐5 decreased pronouncedly with time of incubation. For cow milk yoghurt, the final population for samples with MD‐LA and PWP‐LA was similar at about 6 log cfu/ml, which was much higher than that of control sample (5.36 log cfu/ml). The final population of probiotic population in goat milk yoghurt was 3.51 log cfu/ml for the control samples. It was reported that *L. acidophilus* LA‐5 had an especially poor viability in goat milk yoghurt (Li, Walsh, Gokavi, & Guo, 2012). Compared with control, the final probiotic population was improved to 5.16, 4.32, and 5.13 log cfu/ml by protection using MD, WP, and PWP, respectively.

## 4 CONCLUSIONS

Whey protein with or without heating showed protection effect for *Lactobacillus acidophilus* LA‐5 comparable to maltodextrin after freeze‐drying and during 180‐day storage. Addition of freeze‐dried *L. acidophilus* LA‐5 using polymerized whey protein as protecting material improved the physiochemical and textural properties of cow and goat milk yoghurts compared with maltodextrin. *L. acidophilus* LA‐5 protected by polymerized whey protein incorporated into yoghurt in freeze‐drying microencapsulated form exhibited satisfactory metabolic activity. Data indicated that polymerized whey protein may be a better protecting agent for *Lactobacillus acidophilus* LA‐5 during freeze‐drying compared with maltodextrin by producing better probiotic viability during storage, better yoghurt quality, and only minor difference in survival rate of *L. acidophilus* LA‐5 during freeze‐drying.

## CONFLICT OF INTEREST

The authors declare that they have no conflict of interest.

## ETHICAL APPROVAL

Human and animal testing is not applicable in our study.
